# Optical motion capture dataset of selected techniques in beginner and advanced Kyokushin karate athletes

**DOI:** 10.1038/s41597-021-00801-5

**Published:** 2021-01-18

**Authors:** Agnieszka Szczęsna, Monika Błaszczyszyn, Magdalena Pawlyta

**Affiliations:** 1 Department of Computer Graphics, Vision and Digital Systems, Faculty of Automatic Control, Electronics and Computer Science, Silesian University of Technology, 44-100 Gliwice, Akademicka 16 Poland; 2 Faculty of Physical Education and Physiotherapy, Opole University of Technology, 45-758 Opole, Prószkowska 76 Poland; 3 Polish-Japanese Academy of Information Technology, 02-008 Warsaw, Koszykowa 86 Poland

**Keywords:** Biomedical engineering, Public health

## Abstract

Human motion capture is commonly used in various fields, including sport, to analyze, understand, and synthesize kinematic and kinetic data. Specialized computer vision and marker-based optical motion capture techniques constitute the gold-standard for accurate and robust human motion capture. The dataset presented consists of recordings of 37 Kyokushin karate athletes of different ages (children, young people, and adults) and skill levels (from 4th dan to 9th kyu) executing the following techniques: reverse lunge punch (*Gyaku-Zuki*), front kick (*Mae-Geri*), roundhouse kick (*Mawashi-Geri*), and spinning back kick (*Ushiro-Mawashi-Geri*). Each technique was performed approximately three times per recording (i.e., to create a single data file), and under three conditions where participants kicked or punched (i) in the air, (ii) a training shield, or (iii) an opponent. Each participant undertook a minimum of two trials per condition. The data presented was captured using a Vicon optical motion capture system with Plug-In Gait software. Three dimensional trajectories of 39 reflective markers were recorded. The resultant dataset contains a total of 1,411 recordings, with 3,229 single kicks and punches. The recordings are available in C3D file format. The dataset provides the opportunity for kinematic analysis of different combat sport techniques in attacking and defensive situations.

## Abstract (summary)

**Table:** 

Measurement(s)	3D trajectory • joints angle • joints force • joints power • joints moment • body movement coordination trait
Technology Type(s)	optical motion capture
Factor Type(s)	age • technique • skill level • condition
Sample Characteristic - Organism	Homo sapiens
Sample Characteristic - Environment	laboratory environment

Machine-accessible metadata file describing the reported data: 10.6084/m9.figshare.13164848

## Background & Summary

Human motion capture is commonly used in various fields, including sport, to analyze, understand, and synthesize kinematic and kinetic data.

The ability to execute the right technique in combat sports plays an important role in scoring points. However, there is no common optimal movement pattern for the performance of individual techniques. The martial art of karate is a method of fighting and defending with an “empty hand” (i.e., without a weapon)^1^. Karate requires physical, technical, and tactical skills, and is based on techniques involving striking the opponent with the hand, foot, knee, or elbow. The movement patterns for these striking techniques typically involve flexion, extension, abduction, adduction, and rotation of various joints. Sufficient force must be transferred through the kinetic chain for striking techniques to score points^2,3^.

In recent years, martial arts has increased in popularity, and this has resulted in rule changes. The basic features of sport karate that make it recognizable are point-scoring techniques such as kicks and punches. These techniques can be used in three basic scenarios: (i) to attack, (ii) to intercept an opponent’s strike, and (iii) to counterattack^4^. In karate, it is extremely important to be able to execute techniques and changes of direction at speed, and this requires high levels of coordination and balance. Research in martial arts focuses primarily on injuries^5,6^, psychology^7–9^, biomechanics^10,11^, and perception of health^12^.

Findings from investigations that have analyzed competitive karate are important for planning the training process, and helping to ensure that training adapts to changes in competition rules. In competition, it has been found that punches and kicks account for 89.09% and 8.36% respectively of all techniques used^13^. An analysis using kinematic methods during karate contests revealed that upper limb techniques achieved a higher score compared to lower limb techniques^14,15^. Furthermore, it has been shown that punches are a more dominant technique compared to kicks, which are used less frequently; this is despite rule changes that favor the use of kicks. Punches are less complex, allow greater precision and control, and require less energy expenditure^13,16,17^. Moreover, punches can be executed quickly, and thus have a greater chance of scoring points^14,15^. However, taking into account spectators’ perceptions, punches are not as spectacular as kicks.

Karate kicking techniques include the front kick (*Mae-Geri*), roundhouse kick (*Mawashi-Geri*), hook kick (*Ura-Mawashi-Geri*), and sidekick (*Yoko-Geri*). The roundhouse kick to the opponent’s head (*Mawashi-Geri jodan*) is the most commonly used kicking technique in karate^18^. However, a roundhouse kick to the opponent’s torso (*Mawashi-Geri chudan*) allows more control, and greater protection from the opponent’s strikes, compared to other kicks; therefore, an athlete may opt to use a roundhouse kick to the torso instead of other kicks^13^.

Based on the research cited above, the importance of human motion analysis in combat sports is evident, with both kinematic and kinetic analysis required^19^. Kinematic analysis is necessary to identify the ranges of motion and speeds required when executing different phases of the movement patterns. Anatomical angles are more important, and facilitate comparison of values from different investigations, regardless of the motion capture system used.

For example, motion analysis studies of karate have investigated reaction time and anticipation^20,21^, kicking limb movement patterns^10^, and the development of segmentation techniques^18^. Based on the positions of the reflective markers in previous studies, the most frequently analyzed variables are angular displacement of the hip, knee, ankle, shoulder, elbow, torso, and head. These variables are most often analyzed in the sagittal plane. Other approaches to analysis include the inter-joint coordination index, coefficients of variation, and the symmetry index. These approaches have been used to investigate movement coordination, movement velocity, and the relationship between them^22,23^. Several studies have shown that velocity is the main factor determining performance in karate athletes^15^. A novel method to measure interpersonal synchronization of movement using motion capture data is to detect relevant acceleration peaks for upper and lower limbs, and then establish if they are synchronized. Such a method has been effective in classifying the skill level of karate athletes performing kata^24^. In^25^ the basic multi-joint movement patterns used by karate athletes of different levels (based on experience and skill level) were identified.

Based on the above considerations, we present a comprehensive set of kinematic and kinetic data obtained from recordings of 37 Kyokushin karate athletes. The athletes were of different ages (children, young people, and adults), and of different skill levels as based upon the karate grading system (from 4th dan to 9th kyu). Data^26^ was obtained for the reverse lunge punch (*Gyaku-Zuki*), front kick (*Mae-Geri*), roundhouse kick (*Mawashi-Geri*), and spinning back kick (*Ushiro-Mawashi-Geri*). Every technique was performed three times per recording (resulting in one data file), and under three conditions: (i) kicking or punching the air, (ii) kicking or punching a training shield, and (iii) kicking or punching an opponent. Possible applications of the data obtained are:

- comparison of movement patterns between individual athletes, or groups of athletes^11^, based upon factors such as age, gender, training experience, and karate grade,
- kinematic description and analysis of movement patterns used when executing karate techniques^24^,
- measure personal and interpersonal repetition of movement^24,27^,
- development of virtual reality environments for virtual training^28,29^,
- training and validation of machine learning techniques for the classification, prediction and synthesis of human movement^30,31^,
- development and optimization of methods for teaching karate techniques.

Human movement data regarding gait^32–34^, activities of daily living (ADL)^35,36^, and general sport activities^37^ is publicly available. However, publicly available human movement data regarding the martial arts is limited. In the Physical Activities and Sports category of the Carnegie Mellon University Motion Capture Database (http://mocap.cs.cmu.edu/) the martial arts subcategory contains recordings of only two subjects (motion described as “punch/strike”, “swordplay” and “tai chi”). In the HDM05 repository^38^ the only martial arts related category (“kicking and punching”) contains 17 recordings, but without technical descriptions of the techniques depicted, or information about martial arts where the techniques are used, whilst the KIT Whole-Body Human Motion Database^39,40^ contains only general recordings described as “kick” and “punch”.

Consequently, there is little publicly available human movement data concerning specific martial arts, including karate. There has been an attempt to create an open karate motion capture data repository with seven participants, and recordings of *Shorin-ryu*, *Shotokan*, and *Oyama* styles by inertial sensor based motion capture system^41^.

Additionally, a further dataset described^42,43^ contains motion capture data (synchronized with video and audio recordings) of two katas performed by seven participants with different levels of experience.

The goal of collecting the Martial Arts, Dancing and Sports dataset (MADS) was to provide challenging action sequences for human pose estimation from multi-view or depth data. The ground-truth pose data was captured by optical motion capture system with only 60 Hz. As part of the database, the recordings of two martial arts masters in 6 forms in tai-chi and 6 katas in karate are available^44^.

Next available motion capture dataset UMONS-TAICHI contains Taijiquan martial art gestures that includes 13 classes (relative to Taijiquan techniques) executed by 12 participants of various skill levels. The dataset was captured using two motion capture systems simultaneously: optical motion capture system with frequency 179 Hz, and markerless motion capture system based on depth sensor^45^.

It is important to address the absence of high quality, well described, and publicly available martial arts motion capture data. Any future repository should contain recordings that depict karate athletes of different levels (e.g., grade, experience) executing techniques under various conditions (e.g., defending and attacking against an opponent).

## Methods

The part of presented dataset was used to investigate the three-dimensional kinematics of the front kick (*Mae-Geri*) when executed by Kyokushin karate athletes of different levels under three conditions^11^: (i) a kick in the air, (ii) a kick against a training shield, and (iii) a kick against an opponent.

### Participants

Thirty-seven healthy participants (13 women, 24 men), aged between 10 to 50 years (mean 18 with std 10), took part in the study. Participants trained at the Kyokushin Karate Club (Gliwice or Nysa, Poland). Participant characteristics were: mass 30–118 kg (mean 54.5 with std 19.9), height 142–192 cm (mean 160 with std 12.8), training experience 2–35 years (mean 9.3 with std 8.4), and karate grade (9th kyu - 4th dan) (Table 1). All participants reported no known movement disorders or other health problems that could affect their mobility. Before starting the recordings, each subject was comprehensively informed about the procedure, introduced to the experiment, and informed of any potential risks. We required the participants to sign an informed consent form. Written consent from parents/legal guardians was obtained for any participants who were minors. The study was carried out according to the Helsinki Declaration, and each of the participants gave their written consent to participate in the research. The study was approved by a local bioethics committee, and carried out between March 2017 and April 2017.

**Table 1:** Participant characteristics.

Code	Age [years]	Gender [F|M]	Weight [kg]	Height [cm]	Training time [years]	Karate grade [Kyu|Dan]
B0367	48	M	80	173	34	4 dan
B0368	24	M	68	170	9	1 dan
B0369	50	M	88	182	30	3 dan
B0370	20	M	71	178	10	2 kyu
B0371	22	M	78	172	7	1 kyu
B0372	25	M	74	172	19	1 dan
B0373	13	F	62	168	7	4 kyu
B0374	11	M	42	150	5	7 kyu
B0375	12	F	45	152	8	8 kyu
B0376	10	F	35	143	6	6 kyu
B0377	11	M	44	154	7	9 kyu
B0378	11	M	49	156	7	8 kyu
B0379	12	M	35	145	3	7 kyu
B0380	13	M	30	144	5	5 kyu
B0381	13	M	50	161	6	5 kyu
B0382	24	M	86	182	12	5 kyu
B0383	10	M	36	142	5	8 kyu
B0384	11	M	44	151	5	6 kyu
B0385	11	F	42	150	3	8 kyu
B0386	11	F	45	152	2	9 kyu
B0387	11	F	52	158	5	7 kyu
B0388	14	F	54	157	4	6 kyu
B0389	14	F	52	153	8	4 kyu
B0391	43	M	80	180	4	3 kyu
B0392	26	M	118	192	10	2 kyu
B0393	13	M	40	160	5	6 kyu
B0394	12	M	30	142	5	7 kyu
B0395	14	M	45	158	4	7 kyu
B0396	17	F	47	164	8	4 kyu
B0398	31	F	62	176	18	1 dan
B0399	20	F	70	166	13	2 kyu
B0400	12	M	35	150	3	8 kyu
B0401	12	M	39	152	3	8 kyu
B0402	12	M	34	150	3	7 kyu
B0403	12	M	32	143	4	8 kyu
B0404	29	F	67	163	29	2 dan
B0405	28	F	62	162	28	2 kyu

### Instrumentation

Data was recorded using a motion tracking system (Vicon Motion Systems Limited, Oxford, UK) sampling at 250 Hz. Thirty-nine reflective markers from the Plug-In Gait software full-body marker set were attached to specific anatomical landmarks (according to the Vicon system documentation). In this approach, one marker is placed on each joint (e.g., elbow, ankle, knee). Between adjacent joints there is a further marker placed at different heights on the right and left limbs to distinguish them from each other. Additionally, four markers are used for the pelvis (for the front and back spines), five for the torso (two for the spine at C7 and TH12, one for the shoulder blade, and two for the breastbone), and four for the head. An additional four markers were placed on the training shield. For recordings with an opponent, both the attacker and defender had markers placed on them, resulting in two sets of data (i.e., a set from the attacker, and a set from the defender). Data acquisition was carried out in the Human Motion Lab (HML) at the Research and Development Center of the Polish-Japanese Academy of Information Technology in Bytom, Poland. The system for data acquisition consisted of ten near-infrared (NIR) Vicon MX-T40 cameras with 4 megapixel resolution and 10-bit grayscale, and 10 Vantage V5 cameras with 5 megapixel resolution. The area used for measurement had the shape of an ellipsoidal cylinder, with a height of 3 m, and a base with axes of 6.47 m and 4.2 m.

### Acquisition protocol

Before the execution of the technique was recorded, participants performed a standardized individual warm-up. The warm-up was approximately 2 minutes duration, and predominately consisted of stretching exercises. The athletes had to execute the designated technique in the measurement area. After the markers were placed on the participant, and before they executed the technique, calibration of the motion capture system was carried out according to the standard Vicon protocol. For the calibration, the athlete had to stand in a “T” position by joining their legs and raising their arms to the side.

The reference position (starting stance) for the participants was *kumite no kamae*. This position involved standing with one foot in front of the other, and both heels touching the ground. The lateral distance between the two feet corresponded to the width of the participant’s pelvis. The designated technique was executed with the rear leg, and following execution of the technique participants returned to the reference position. Participants were instructed to use their dominant leg to execute the technique, with the exception of the opponent condition (i.e., when kicking an opponent), where participants could use either leg to execute the technique in a manner best suited to the combat situation, and their preferred attacking strategy. Participants were instructed to execute the technique with maximum speed, and the intent to achieve maximum force upon impact. No prompt was given to the athletes to start the kick. Participants performed three repetitions of the designated technique, and two trials of each condition were conducted.

**Fig. 1:**
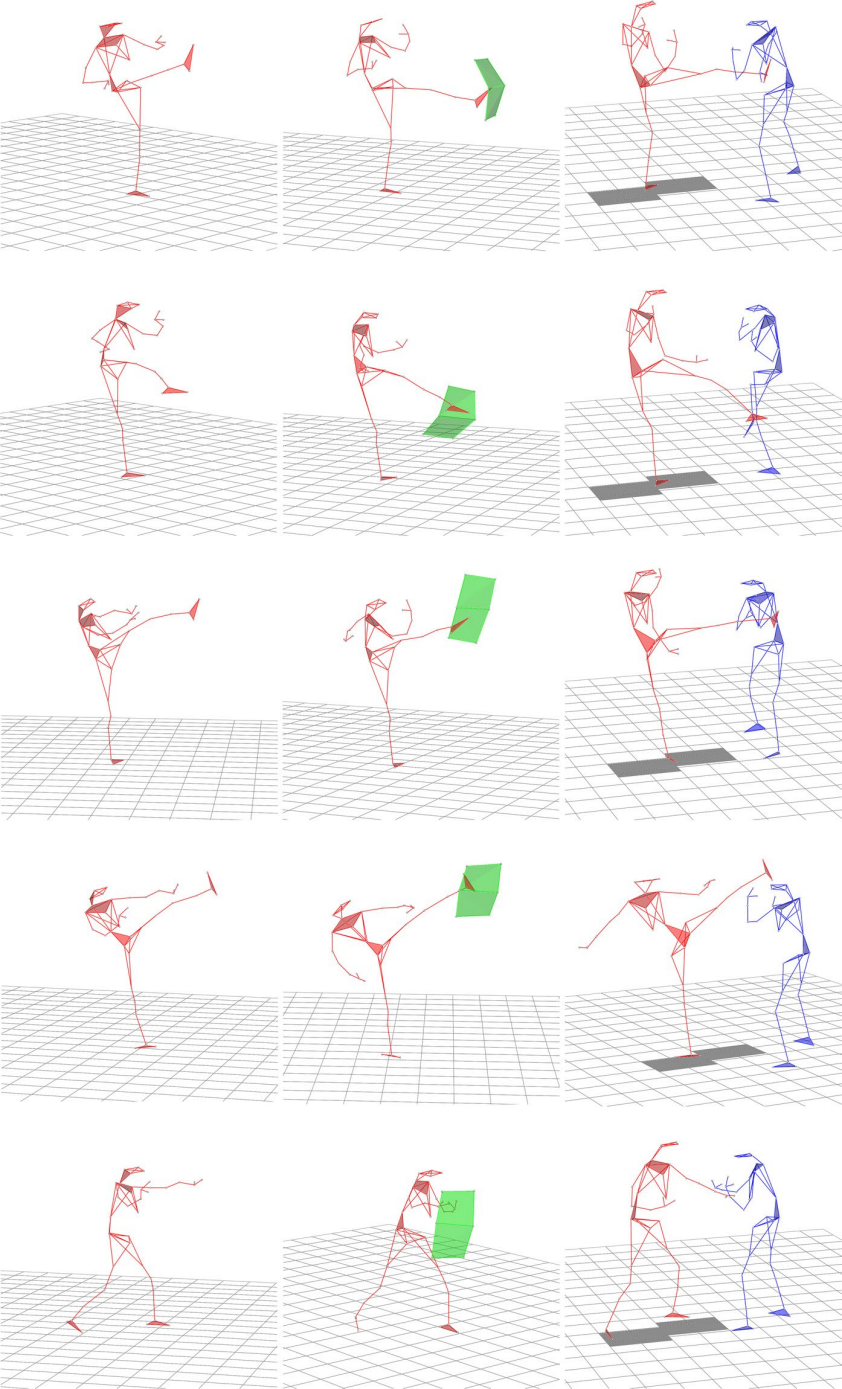
Skeletons (the attacker is represented as a red skeleton) during the following techniques (in rows from the top): *Mae-Geri*, *Mawashi-Geri gedan*, *Mawashi-Geri jodan*, *Ushiro-Mawashi-Geri* and *Gyaku-Zuki*. Three different conditions (in columns from left): air, shield, opponent.
- The roundhouse kick (*Mawashi-Geri*) is also referred to as a round kick. The roundhouse kick is similar to the front kick, the only difference being that the movement pattern for the roundhouse kick is circular, and attacks the opponent from the side. From the fighting stance the kick is executed with the rear leg. The roundhouse kick was recorded at two heights: (i) from knee to hip (*gedan*, Fig. 1, second row), and (ii) from shoulder to the top of the head (*jodan*, Fig. 1, third row). Initially, the joint movements involved in the execution of this technique are flexion, abduction, and external rotation of the hip, and knee flexion, followed by hip internal rotation and extension, and knee extension in the direction of the target.
- The spinning back kick (*Ushiro-Mawashi-Geri*) begins with the fighting stance, and is generally executed with the rear leg (Fig. 1, fourth row). The attacker firstly spins 180 degrees, resulting in their back facing the target. Whilst turning, the knee is brought up such that the angle between the thigh and the calf is 90 degrees. Once in this position, the kick is ready to be executed by extending the leg to strike the target.
- The reverse punch (*Gyaku-Zuki*) is executed with the hand contralateral to the front leg (Fig. 1, fifth row). The objective is to execute the punch quickly, and from a controlled distance. This punch is the first to be learnt due to its simplicity. By executing *Gyaku-Zuki* at speed, the ability of the opponent to anticipate and react is limited. Execution of the punch requires a proximal-to-distal generation of force, beginning at the pelvis, and progressing through the torso and upper arm, before culminating at the fist. The movement begins with rotation of the pelvis, and continues with arm flexion, immediately followed by forearm extension.

Description of recorded techniques:

- Front kick (*Mae-Geri*) is a basic kick. It is useful for self-defense situations such as kicking the opponent (Fig. 1, first row). This kick is usually performed by the rear leg in the fighting stance. The front kick is the most frequently used kick, as it can be performed at speed, requires little preparatory movement, and is difficult to block. There are slight variations in how to perform a front kick, from a quick snap kick (i.e., short contact time) to a powerful thrusting front kick (i.e., longer contact time that “pushes” the opponent away).

The following three conditions were specified:

- a training kick or punch in the air,
- a kick or punch at a target (i.e., a training shield held by the coach),
- a kick or punch against an opponent in a combat situation, with both attacker and defender recorded.

Not all techniques were performed by all participants. For example, the roundhouse kick (*Mawashi-Geri*) and spinning back kick (*Ushiro-Mawashi-Geri*) are technically difficult, and some of the less experienced participants were not able to execute these techniques successfully. If a less experienced participant was unable to execute a technique successfully, there is no recording of the technique in the participant’s catalogue. The overall statistics are listed in Table 2.

**Table 2:** Recording statistics for individual techniques in conditions: air (A), shield (S), opponent (O).

Code	*Mae-Geri*	*Mawashi-Geri Gedan*	*Mawashi-Geri Jodan*	*Ushiro-Mawashi-Geri*	*Gyaku-Zuki*
B0367	6(A),5(S),0(O)	6(A),6(S),0(O)	6(A),6(S),0(O)	6(A),3(S),0(O)	3(A),6(S),0(O)
B0368	6(A),6(S),0(O)	6(A),6(S),0(O)	6(A),6(S),0(O)	6(A),6(S),0(O)	9(A),6(S),0(O)
B0369	6(A),6(S),0(O)	6(A),6(S),0(O)	6(A),6(S),0(O)	6(A),6(S),0(O)	6(A),6(S),0(O)
B0370	6(A),6(S),0(O)	6(A),6(S),0(O)	6(A),6(S),0(O)	0(A),0(S),0(O)	6(A),6(S),0(O)
B0371	6(A),6(S),6(O)	6(A),6(S),6(O)	6(A),6(S),6(O)	6(A),6(S),6(O)	6(A),6(S),6(O)
B0372	6(A),6(S),6(O)	6(A),6(S),6(O)	6(A),6(S),6(O)	6(A),6(S),6(O)	6(A),6(S),6(O)
B0373	6(A),6(S),6(O)	6(A),6(S),6(O)	6(A),6(S),6(O)	6(A),6(S),6(O)	6(A),6(S),6(O)
B0374	6(A),6(S),6(O)	6(A),6(S),6(O)	6(A),6(S),6(O)	6(A),6(S),6(O)	6(A),6(S),8(O)
B0375	6(A),6(S),6(O)	6(A),6(S),6(O)	6(A),6(S),6(O)	6(A),6(S),6(O)	6(A),6(S),7(O)
B0376	6(A),6(S),3(O)	6(A),6(S),8(O)	6(A),6(S),6(O)	9(A),6(S),6(O)	6(A),6(S),7(O)
B0377	6(A),6(S),6(O)	6(A),6(S),6(O)	6(A),6(S),6(O)	6(A),6(S),6(O)	6(A),6(S),6(O)
B0378	6(A),6(S),6(O)	6(A),6(S),6(O)	6(A),9(S),6(O)	6(A),3(S),6(O)	6(A),6(S),6(O)
B0379	7(A),6(S),6(O)	6(A),6(S),6(O)	6(A),6(S),6(O)	6(A),7(S),6(O)	6(A),7(S),11(O)
B0380	6(A),6(S),6(O)	6(A),6(S),6(O)	6(A),6(S),6(O)	6(A),8(S),6(O)	6(A),7(S),6(O)
B0381	6(A),6(S),9(O)	6(A),6(S),6(O)	6(A),6(S),5(O)	6(A),6(S),6(O)	6(A),6(S),7(O)
B0382	6(A),6(S),0(O)	6(A),6(S),0(O)	6(A),6(S),0(O)	6(A),6(S),0(O)	7(A),6(S),0(O)
B0383	6(A),6(S),0(O)	3(A),6(S),0(O)	6(A),6(S),0(O)	6(A),6(S),0(O)	6(A),6(S),0(O)
B0384	6(A),6(S),9(O)	6(A),6(S),6(O)	6(A),6(S),6(O)	6(A),6(S),6(O)	6(A),6(S),8(O)
B0385	6(A),6(S),6(O)	6(A),6(S),6(O)	6(A),6(S),6(O)	6(A),6(S),8(O)	6(A),6(S),10(O)
B0386	6(A),6(S),7(O)	6(A),6(S),6(O)	6(A),6(S),9(O)	6(A),6(S),6(O)	6(A),6(S),8(O)
B0387	6(A),6(S),6(O)	6(A),6(S),6(O)	6(A),6(S),6(O)	6(A),6(S),6(O)	6(A),6(S),6(O)
B0388	6(A),6(S),12(O)	6(A),6(S),12(O)	6(A),6(S),11(O)	6(A),6(S),12(O)	6(A),6(S),12(O)
B0389	6(A),6(S),6(O)	6(A),6(S),6(O)	5(A),6(S),6(O)	6(A),6(S),6(O)	6(A),6(S),6(O)
B0391	6(A),6(S),6(O)	6(A),6(S),6(O)	6(A),6(S),6(O)	6(A),6(S),6(O)	6(A),8(S),6(O)
B0392	6(A),6(S),6(O)	6(A),6(S),6(O)	6(A),6(S),6(O)	6(A),6(S),6(O)	6(A),6(S),7(O)
B0393	7(A),6(S),6(O)	6(A),9(S),7(O)	6(A),6(S),6(O)	6(A),6(S),6(O)	6(A),18(S),7(O)
B0394	6(A),6(S),6(O)	6(A),6(S),8(O)	6(A),6(S),6(O)	6(A),6(S),6(O)	6(A),6(S),7(O)
B0395	6(A),6(S),6(O)	6(A),6(S),6(O)	6(A),6(S),6(O)	6(A),6(S),6(O)	6(A),6(S),9(O)
B0396	6(A),6(S),6(O)	6(A),6(S),6(O)	6(A),6(S),6(O)	6(A),6(S),6(O)	6(A),6(S),6(O)
B0398	6(A),6(S),6(O)	6(A),6(S),6(O)	6(A),6(S),6(O)	6(A),6(S),6(O)	9(A),6(S),6(O)
B0399	6(A),6(S),6(O)	6(A),6(S),6(O)	6(A),6(S),6(O)	6(A),6(S),3(O)	6(A),6(S),6(O)
B0400	6(A),6(S),7(O)	6(A),6(S),7(O)	6(A),6(S),7(O)	6(A),6(S),7(O)	7(A),6(S),7(O)
B0401	6(A),6(S),6(O)	6(A),6(S),6(O)	6(A),6(S),6(O)	6(A),3(S),6(O)	6(A),6(S),6(O)
B0402	3(A),6(S),6(O)	6(A),6(S),6(O)	6(A),6(S),6(O)	6(A),6(S),6(O)	6(A),6(S),6(O)
B0403	6(A),6(S),6(O)	6(A),6(S),6(O)	6(A),6(S),9(O)	6(A),6(S),6(O)	6(A),5(S),6(O)
B0404	6(A),6(S),6(O)	6(A),6(S),6(O)	6(A),6(S),6(O)	6(A),6(S),6(O)	6(A),6(S),6(O)
B0405	6(A),6(S),6(O)	6(A),6(S),6(O)	6(A),6(S),6(O)	6(A),6(S),6(O)	6(A),6(S),6(O)

Figure 1 shows single frame images of skeletons (represented by stick figures with joint markers) performing each technique under each condition. The techniques were performed as described above.

### Data preprocessing and available variables

Plug-In Gait software was used to determine angles, moments, force output, and power output at individual joints, and to estimate virtual markers, such as the center of mass (COM). A description of all variables is available in the system documentation (https://docs.vicon.com). The data in the repository is non-normalized giving the broadest possibility of analysis. Available data contains 3D trajectories of all markers (set of 39 markers) and angles of human joints without information about the skeleton. Additional moments, powers and forces in those joints. There are also trajectories of shield markers for determining the position of the target. The additional four trajectory markers used on the training shield are labeled as: *Tarcza1*, *Tarcza2*, *Tarcza3* and *Tarcza4*.

## Data Records

### Dataset organization

In the dataset, there is one catalogue for each participant (37 catalogues in total). Catalogues are named as per the participant code number (see Table 1). Each catalogue contains sub-folders corresponding to the techniques recorded. Sub-folders are labeled as follows:

YYYY-MM-DD-CODE-S0X

where

YYYY - year

MM - month

DD - day

CODE - participant code;

S0X - karate technique, S01 - *Gyaku-Zuki*, S02 - *Mae-Geri*, S03 - *Mawashi-Geri gedan*, S04 - *Mawashi-Geri jodan*, S05 - *Ushiro-Mawashi-Geri*.

The following file labeling convention is used:

YYYY-MM-DD-CODE-S0X-E0Z-T0J.c3d

where

E0Z - condition: E01 - air, E02 - shield, E03 - attacker, E04 - defender;

T0X - number for trial, T01 or T02.

The dataset comprises 1,411 files, with 3,229 single kicks and punches. Data is stored in the C3D file format (https://www.c3d.org/). There are 3–4 repetitions of the same technique in a given trial (T01 and T02). It gives also the possibility of a time and a preparatory movement analysis for the technique. The C3D file format is widely used in the biomechanical field by companies and laboratories to store motion capture system data. The dataset is available at figshare (10.6084/m9.figshare.c.4981073)^26^.

## Technical Validation

Normalization was required for analysis of the data. Here a very basic normalization is proposed concerning only the analysis on the basis of one selected ankle maker for kicks and a finger for the punching. The data gives the possibility of a much broader and comprehensive analysis using a full-body set of information. The time taken to execute the same technique differed between and within subjects. Therefore, the data was normalized for time. Using an approach taken from gait analysis research, the start and end points for a given technique were determined, and then scaled to ensure that execution of the technique always lasted a given number of frames. The algorithm used consisted of several steps. The first step was to increase the sampling frequency by a given value by adding zeros to the signal. Then the finite impulse response (FIR) anti-aliasing filter was applied. The last step was to downsample the filtered signal to the desired value by discarding the samples. The trajectories obtained in this way had the same length, whilst maintaining their shape. These steps were carried out using the *resample* function available in Matlab.

**Fig. 2:**
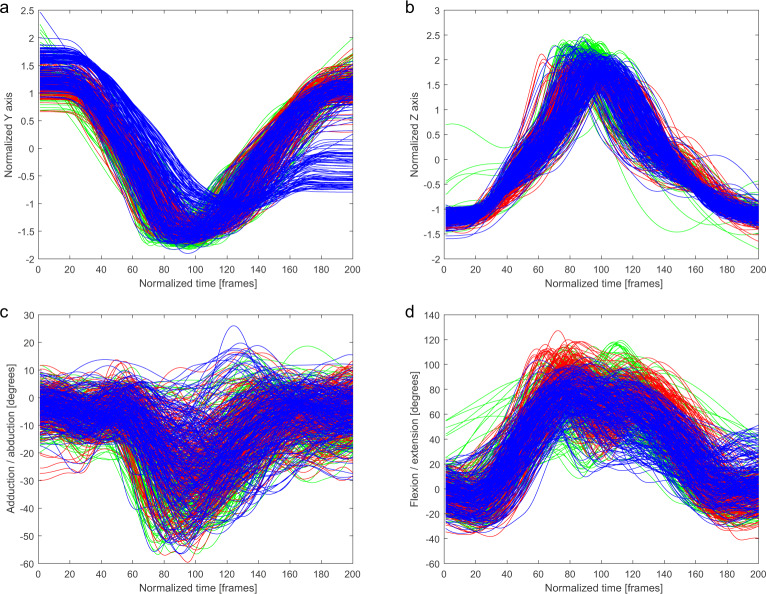
Ankle marker trajectory and hip joint angles of the kicking leg in *Mae-Geri* technique for three conditions: air (green), shield (red), opponent (blue). (**a**) Normalized y coordinate of trajectory of the kicking leg ankle marker. (**b**) Normalized z coordinate of trajectory of the kicking leg ankle marker. (**c**) Adduction/abduction hip angle of the kicking leg. (**d**) Flexion/extension hip angle of the kicking leg.

**Fig. 3:**
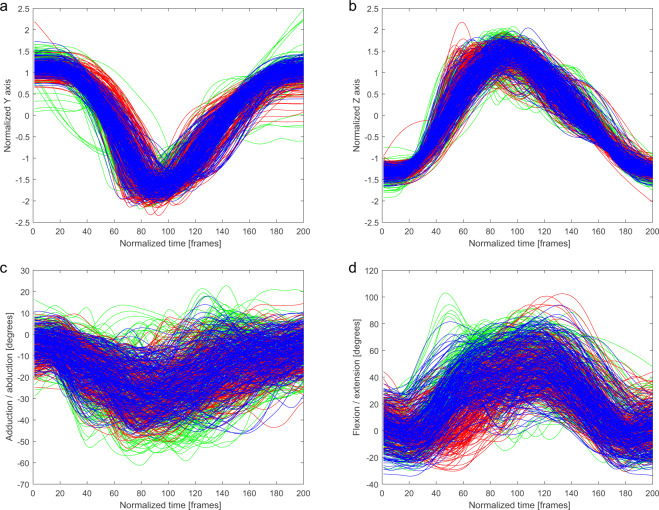
Ankle marker trajectory and hip joint angles of the kicking leg in *Mawashi-Geri gedan* technique for three conditions: air (green), shield (red), opponent (blue). (**a**) Normalized y coordinate of trajectory of the kicking leg ankle marker. (**b**) Normalized z coordinate of trajectory of the kicking leg ankle marker. (**c**) Adduction/abduction hip angle of the kicking leg. (**d**) Flexion/extension hip angle of the kicking leg.

**Fig. 4:**
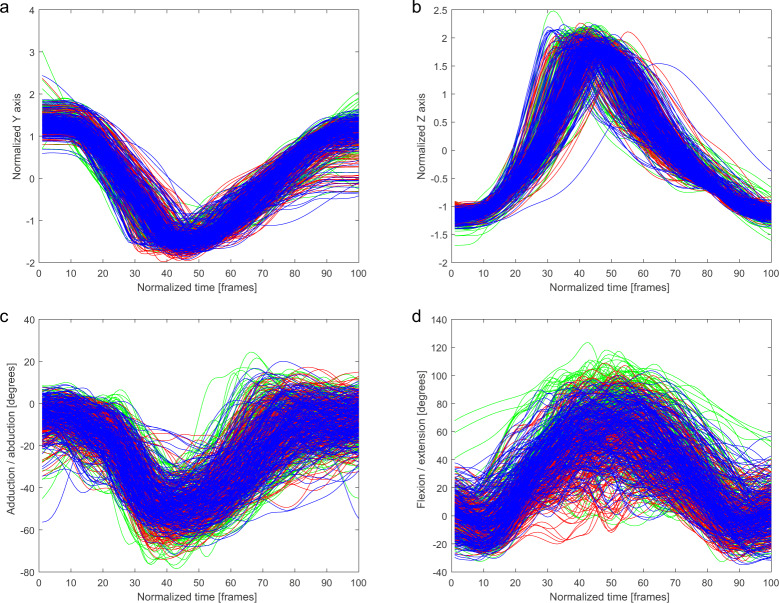
Ankle marker trajectory and hip joint angles of the kicking leg in *Mawashi-Geri jodan* technique for three conditions: air (green), shield (red), opponent (blue). (**a**) Normalized y coordinate of trajectory of the kicking leg ankle marker. (**b**) Normalized z coordinate of trajectory of the kicking leg ankle marker. (**c**) Adduction/abduction hip angle of the kicking leg. (**d**) Flexion/extension hip angle of the kicking leg.

**Fig. 5:**
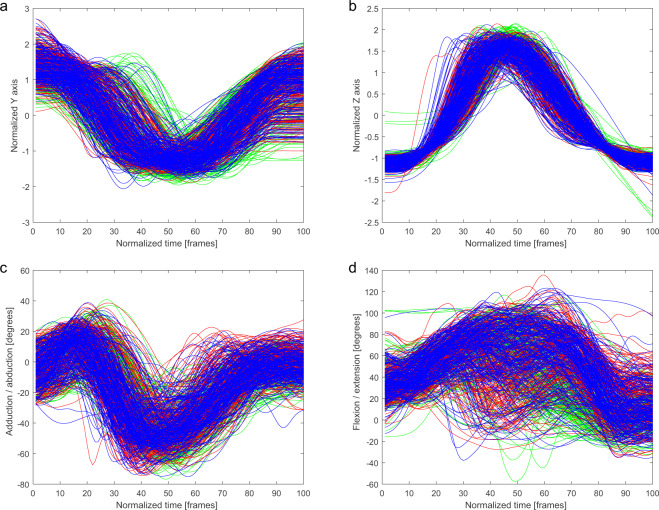
Ankle marker trajectory and hip joint angles of the kicking leg in *Ushiro-Mawashi-Geri* technique for three conditions: air (green), shield (red), opponent (blue). (**a**) Normalized y coordinate of trajectory of the kicking leg ankle marker. (**b**) Normalized z coordinate of trajectory of the kicking leg ankle marker. (**c**) Adduction/abduction hip angle of the kicking leg. (**d**) Flexion/extension hip angle of the kicking leg.

**Fig. 6:**
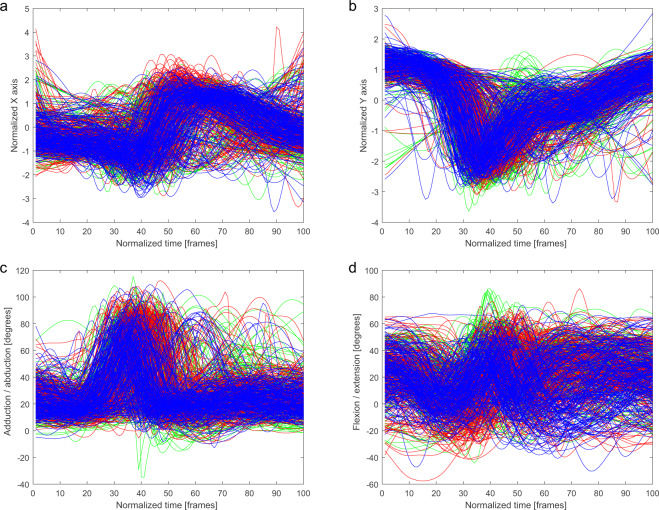
Finger marker trajectory and shoulder joint angles of the punching upper limb in *Gyaku-Zuki* technique for three conditions: air (green), shield (red), opponent (blue). (**a**) Normalized x coordinate of trajectory of the punching upper limb finger marker. (**b**) Normalized y coordinate of trajectory of the punching upper limb finger marker. (**c**) Adduction/abduction shoulder angle of the punching upper limb. (**d**) Flexion/extension shoulder angle of the punching upper limb.

For joint angles, moments, force outputs, and power outputs, this normalization was sufficient. However, for the trajectory of joint markers spatial normalization was necessary. The participant’s position within the scene affected the marker position (e.g., the participant’s height affected their kick height). Therefore, all trajectories had be normalized in some way. A basic method to standardize trajectories is to use the z-score of *p* (the time series of coordinates for x, y, and z):

1 $$z=\frac{(p-\mu )}{\sigma }$$

where *μ* is the mean value and σ is the standard deviation of p. This method has been used for motion feature normalization in classification tasks^46,47^. Figures 2 to 6 were normalized using this method.

Whilst this method works well for simple visualization, it is too simple to evaluate individual trajectories. For example, this method of normalizing would not show the difference in kick height. To obtain such information, other normalization methods that take into consideration factors such as participants’ limb length and height can be used.

When analyzing movement anatomical axis and planes are used. The x-axis is the frontal axis (representing movement from the left to right side of the body), the y-axis is the sagittal axis (representing front and back movements), and the z-axis is the vertical axis (representing up and down movements).

To automatically detect the kick, and the kicking leg (right or left), the value of the z coordinate and basic peak analysis can be used. If the peak value exceeds a specified limit, it means a kick has occurred. To standardize the kicking leg data (e.g., the left leg), the value of the x coordinate should be changed to the opposite side (i.e., reflection transformation).

The trajectories of the ankle marker on the kicking leg are presented on the charts. Each condition has been drawn using a different color: (i) a kick in the air (green), (ii) a kick at the training shield (red), and (iii) a kick at the opponent (blue). Kicking leg hip joint angles, punching upper limb finger marker trajectories, and punching upper limb shoulder angles are presented in the same manner.

In Figs. 2–6, a similarity in the trajectories and angles observed in participants for a given technique can be seen. The movement is presented from the preparatory phase to the final phase. Therefore, it is possible to divide the movement into its composite phases, and to select specific phases for comparative analysis.

For the *Mae-Geri* kick (Fig. 2), the ankle marker trajectory is presented in the sagittal plane (Fig. 2a), and as vertical axis coordinates (Fig. 2b). The angle value ranges show that the movement takes place mainly in the frontal and sagittal planes (Fig. 2c,d), with the large ranges (-20 degrees to 120 degrees) in Fig. 2d dependent on the phase of the movement. The *Mawashi-Geri gedan* (Fig. 3) is a sidekick, with the largest range of motion seen in the sagittal (Fig. 3d) and frontal (Fig. 3c) planes. The ankle marker trajectory shows the high repeatability of the technique for individual participants. Figure 4 presents the same kick but at a much higher height, as shown by the ankle marker ranges. Figure 5 presents the ankle marker trajectory and hip joint angles for the spinning back kick. The range of hip joint angles is large, but compared to other kicking techniques, participants found it difficult to achieve the required range of motion. Additionally, movement phases can be observed in the upper limb techniques (Fig. 6) for the finger marker (Fig. 6a,b) and shoulder joint angle (Fig. 6c,d).

## Usage Notes

The dataset can be used for kinematic analyses. The Biomechanical ToolKit (BTK)^48^, or standalone application Mokka, can be used to read and visualize the C3D files. BTK and Mokka also allow data to be exported from C3D to other file types (e.g., comma-separated values; CSV). For analyses in Matlab, the external MoCap Toolbox can be used to open C3D files. Data normalization and synchronization appropriate to the planned analysis are required^43,49^.

## Data Availability

Matlab version 2020a was used for all analyses. An external MoCap Toolbox (version 1.5) is required to open C3D files (the Toolbox can be downloaded from https://www.jyu.fi/hytk/fi/laitokset/mutku/en/research/materials/mocaptoolbox). The code used is available upon request.
